# Maize tassels detection: a benchmark of the state of the art

**DOI:** 10.1186/s13007-020-00651-z

**Published:** 2020-08-08

**Authors:** Hongwei Zou, Hao Lu, Yanan Li, Liang Liu, Zhiguo Cao

**Affiliations:** 1 Key Laboratory of Image Processing and Intelligent Control, School of Artificial Intelligence and Automation, Huazhong University of Science and Technology, Wuhan, 430074 China; 2 School of Computer Science and Engineering, Wuhan Institute of Technology, Wuhan, 430205 China

**Keywords:** Maize tassels, Object detection, Object counting, Computer Vision, Deep learning, Convolutional neural networks

## Abstract

**Background:**

The population of plants is a crucial indicator in plant phenotyping and agricultural production, such as growth status monitoring, yield estimation, and grain depot management. To enhance the production efficiency and liberate labor force, many automated counting methods have been proposed, in which computer vision-based approaches show great potentials due to the feasibility of high-throughput processing and low cost. In particular, with the success of deep learning, more and more deeper learning-based approaches are introduced to deal with agriculture automation. Since different detection- and regression-based counting models have distinct characteristics, how to choose an appropriate model given the target task at hand remains unexplored and is important for practitioners.

**Results:**

Targeting in-field maize tassels as a representative case study, the goal of this work is to present a comprehensive benchmark of state-of-the-art object detection and object counting methods, including Faster R-CNN, YOLOv3, FaceBoxes, RetinaNet, and the leading counting model of maize tassels—TasselNet. We create a Maize Tassel Detection Counting (MTDC) dataset by supplementing bounding box annotations to the Maize Tassels Counting (MTC) dataset to allow the training of detection models. We investigate key factors effecting the practical applications of the models, such as convergence behavior, scale robustness, speed-accuracy trade-off, as well as parameter sensitivity. Based on our benchmark, we summarise the advantages and limitations of each method and suggest several possible directions to improve current detection- and regression-based counting approaches to benefit next-generation intelligent agriculture.

**Conclusions:**

Current state-of-the-art detection- and regression-based counting approaches can all achieve a relatively high degree of accuracy when dealing with in-field maize tassels, with at least 0.85 $R^2$ values and 28.2% *rRMSE* error. While detection-based methods are more robust than regression-based methods in scale variations and can infer extra information (e.g., object positions and sizes), the latter ones have significantly faster convergence behaviors and inference speed. To choose an appropriate in-filed plant counting method, accuracy, robustness, speed and some other algorithm-specific factors should be taken into account with the same priority. This work sheds light on different aspects of existing detection and counting approaches and provides guidance on how to tackle in-field plant counting. The MTDC dataset is made available at https://git.io/MTDC

## Background

Extracting key information from images and videos with computer vision techniques is of significant importance for plants phenotyping [1]. There are numerous applications using computer vision technologies in agricultural automation, such as disease detection [2, 3], weeds identification [4, 5], yield estimation [6–8], characterization [9, 10], as well as continuous monitoring of crop growth status [11]. In these applications, plant counting plays a crucial role because it can not only reflect growth status [12, 13] but also be a good indicator of crop yield. Growth status can help analyse the relationship between field management and agrometeorological conditions to provide effective agricultural guidance [14], and knowing crops growth status allows growers to appropriately time field operations, such as fertilization, irrigation, cultivation, etc., which significantly improves yields [15]. In this paper, we focus on this challenging task with state-of-the-art computer vision techniques.

A typically common practice to address plant counting is manual counting with a large number of crews. This is laborious, error-prone, costly and inefficient. More importantly, the need of large-scale and high-throughput analyses in modern agriculture makes it impossible to deal with such tasks in a manual manner. To alleviate this situation, many image-based approaches have been proposed in recent years. Li et al. [16] proposed to detect, count and measure the geometric properties of spikes of a plant grown in controlled glasshouse with neural networks and Laws texture energy. Aich et al. [17] adopted a deep convolutional network to directly predict the number of rosette leaves in a data-driven way. Praveen [18] proposed a graph-based model by exploiting brightness distribution, color feature and circular Hough transform [19]. Considering the gap between controlled environment and field conditions, Lu et al. [6] proposed to count maize tassels under unconstrained field-based environment and introduced a deep convolutional neural network (CNN)-based local count regression framework as well as a Maize Tassel Counting (MTC) dataset. By contrast, Rahnemoonfar et al. [20] attempted to directly regress the global count of fruits with a CNN model, Hasan et al. [8] first employed R-CNN [21] for spike detection with wheat images taken in the field, and Madec et al. [7] considered tackling ear detection from high-resolution RGB imagery with Faster R-CNN [22]. Since plant images with corresponding phenotypic labels are hard to collect and annotate, Ubbens et al. [23] introduced a new method for augmenting plant phenotyping datasets using rendered images of syntheric plants and demonstrated that it can improve performance on the leaf counting task. Moreover, to facilitate image-based techniques for plants phenotyping, five sessions of *Leaf Counting Challenge* were held in conjunction with *Computer Vision Problems in Plant Phenotyping* (CVPPP) workshops from 2014 to 2019 [24–28].

With methods mentioned above, impressive progress has been made in plant counting in recent years. Plant counting, however, remains a known challenging task in the field of plant science and agriculture. Compared to the rapid development of object counting and object detection in Computer Vision communities, efforts made for crop counting seem limited, and the progress is lagged. Existing methods in plant counting generally can be categorized into two paradigms. One is to directly regress the plant counts by resorting to a regression model. The other is to detect individual plants with object detection methodologies [21, 22]. Both paradigms have merits and drawbacks. Despite regression-based methods, e.g., TasselNet [6], can achieve state-of-the-art performance with light-weight computation costs, they can only estimate the count and approximate the distribution of plants. Detection-based methods, by contrast, can infer counts, positions as well as sizes of plants, but the accuracy is not comparable to regression-based ones, especially when dealing with congested scenes, and the model capacity and computational costs are usually large and expensive. How to choose an appropriate paradigm given the target task at hand sometimes is hard for practitioners specializing in plant science and agriculture.

Targeting in-field maize plants, a representative agricultural crop, the goal of this work is to present a comprehensive evaluation of state-of-the-art object detection and object counting methods on the task of maize tassels counting. Object detection is a typical dense prediction problem [29, 30]. In recent years, there appear many advanced object detection approaches, such as R-CNN [21], Fast R-CNN [31], Faster R-CNN [22], SSD [32], YOLO9000 [33], RetinaNet [34], etc. Here we evaluate two one-stage detectors, i.e., RetinaNet [34] and YOLOv3 [35], a widely-used two-stage detector Faster R-CNN [22] and a light-weight face detector FaceBoxes [36]. Different from the original VGG16-based [37] Faster R-CNN, we adopt the feature pyramid network (FPN) [38] to extract features (details about these methods are introduced in the "Methods" section). Furthermore, inspired by TasselNet [6], we construct $\hbox {TasselNet}^*$ by updating the backbone of TasselNet with ResNet34 [39]. We then make a comprehensive evaluation of these methods in the hope that our evaluation can help agriculturist, plant scientists, biologists and breeders choose an appropriate counting paradigm according to their task requirements.

For a fair comparison, a Maize Tassel Detection and Counting (MTDC) dataset is constructed by adding bounding box annotations to the released MTC [6] dataset. We compare counting performance, scale robustness, speed and some other characteristics of considered methods via extensive experiments on the MTDC dataset. According to the experimental results, we summarize our evaluations and suggest some possible solutions to deal with in-field counting tasks.

Overall, the contributions of this paper are two-fold:

- A systematic evaluation of state-of-the-art object detection and regression-based counting approaches on the task of maize tassels counting;
- The MTDC dataset: a maize tassel detection dataset constructed by adding bounding box annotations to the challenging MTC dataset.

## Methods

This section introduces the proposed maize tassel detection and counting dataset, as well as four state-of-the-art detection algorithms and the leading tassel counting methods.

### Maize tassels detection and counting dataset

To evaluate the state-of-the-art methods on maize tassel detection, we re-annotate the MTC dataset [6] with bounding boxes instead of dotted annotations.

The MTC dataset includes 361 images which are randomly chosen from 16 independent time series image sequences, covering from tasselling stage to flowering stage. These sequences are collected from 4 different experimental fields across China between 2010 and 2015 with high-resolution CCD digital camera (E450 Olympus). The row spacing is 25–30 cm, and line spacing is 50–60 cm. Six cultivars of maize plants are involved. Images are taken from a 5-meters-height (4 m for Gucheng sequences) camera whose pose is 60 degree relative to the vertical direction. The original image resolutions for Zhengzhou, Gucheng, Jalaid sequences are $3648 \times 2736$, $4272 \times 2848$, $3456 \times 2304$, respectively. A focal length of 16 mm is fixed to photograph the fields, and the field of view is about 30 $m^2$. The MTC dataset is split into two parts: 186 images for training and validation, 175 images for testing. Among them, images from the training set and validation set come from identical sequences, and images from the test set come from different sequences (as summarised in Table 1). Moreover, the dotted annotations for each image are provided as well.

**Table 1:** Training set (train), validation set (val) and test set (test) settings of the MTC dataset

Sequence	*Num*	Location	Cultivar	train	val	test
Zhengzhou2010	37	China, $34.7^{\circ }$ latitude North, $113.6^{\circ }$ longitude East	Jundan No.20	$\checkmark$	$\checkmark$	
Zhengzhou2011	24	China, $34.7^{\circ }$ latitude North, $113.6^{\circ }$ longitude East	Jundan No.20			$\checkmark$
Zhengzhou2012	22	China, $34.7^{\circ }$ latitude North, $113.6^{\circ }$ longitude East	Zhengdan No.958	$\checkmark$	$\checkmark$	
Taian2010_1	30	China, $36.1^{\circ }$ latitude North, $117.1^{\circ }$ longitude East	Wuyue No.3	$\checkmark$	$\checkmark$	
Taian2010_2	32	China, $36.1^{\circ }$ latitude North, $117.1^{\circ }$ longitude East	Wuyue No.3			$\checkmark$
Taian2011_1	21	China, $36.1^{\circ }$ latitude North, $117.1^{\circ }$ longitude East	Nongda No.108	$\checkmark$	$\checkmark$	
Taian2011_2	19	China, $36.1^{\circ }$ latitude North, $117.1^{\circ }$ longitude East	Nongda No.108			$\checkmark$
Taian2012_1	41	China, $36.1^{\circ }$ latitude North, $117.1^{\circ }$ longitude East	Zhengdan No.958	$\checkmark$	$\checkmark$	
Taian2012_2	23	China, $36.1^{\circ }$ latitude North, $117.1^{\circ }$ longitude East	Zhengdan No.958			$\checkmark$
Taian2013_1	8	China, $36.1^{\circ }$ latitude North, $117.1^{\circ }$ longitude East	Zhengdan No.958	$\checkmark$	$\checkmark$	
Taian2013_2	8	China, $36.1^{\circ }$ latitude North, $117.1^{\circ }$ longitude East	Zhengdan No.958			$\checkmark$
Gucheng2012	15	China, $39.1^{\circ }$ latitude North, $115.7^{\circ }$ longitude East	Jidan No.32	$\checkmark$	$\checkmark$	
Gucheng2014	45	China, $39.1^{\circ }$ latitude North, $115.7^{\circ }$ longitude East	Zhengdan No.958			$\checkmark$
Jalaid2015_1	12	China, $46.7^{\circ }$ latitude North, $112.9^{\circ }$ longitude East	Tianlong No.9	$\checkmark$	$\checkmark$	
Jalaid2015_2	12	China, $46.7^{\circ }$ latitude North, $112.9^{\circ }$ longitude East	Tianlong No.9			$\checkmark$
Jalaid2015_3	12	China, $46.7^{\circ }$ latitude North, $112.9^{\circ }$ longitude East	Tianlong No.9			$\checkmark$

*Num* refers to the number of images in each sequence

**Fig. 1:**
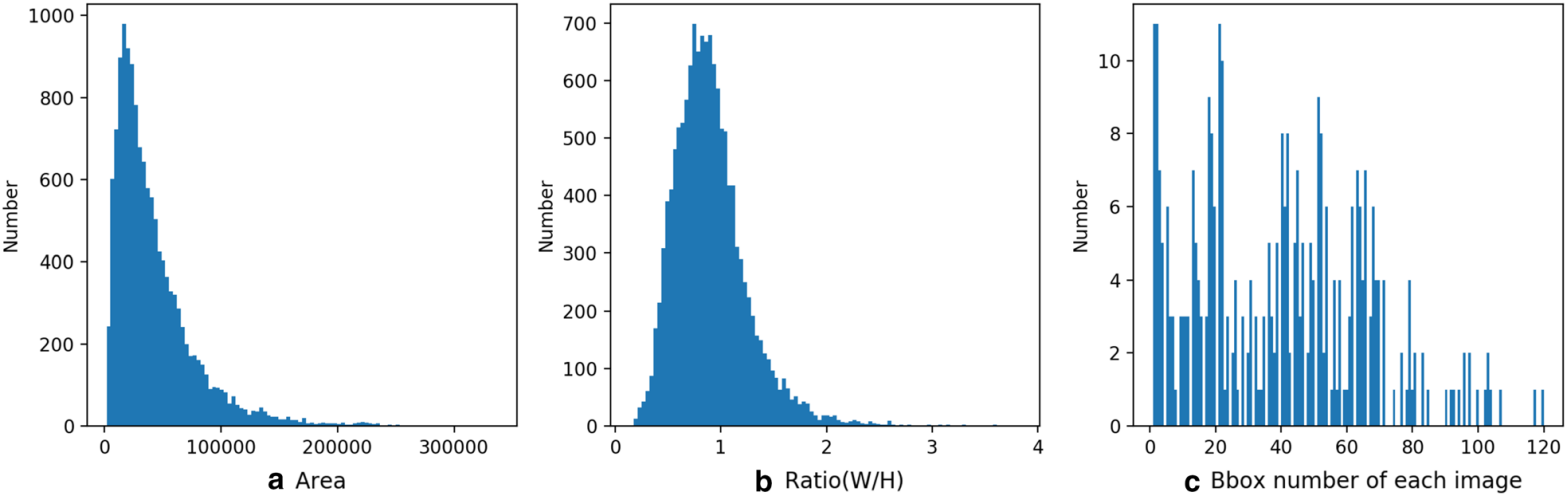
Statistics of MTDC dataset: **a** bbox area; **b** bbox ratio (W/H); **c** bbox number of each image

**Fig. 2:**
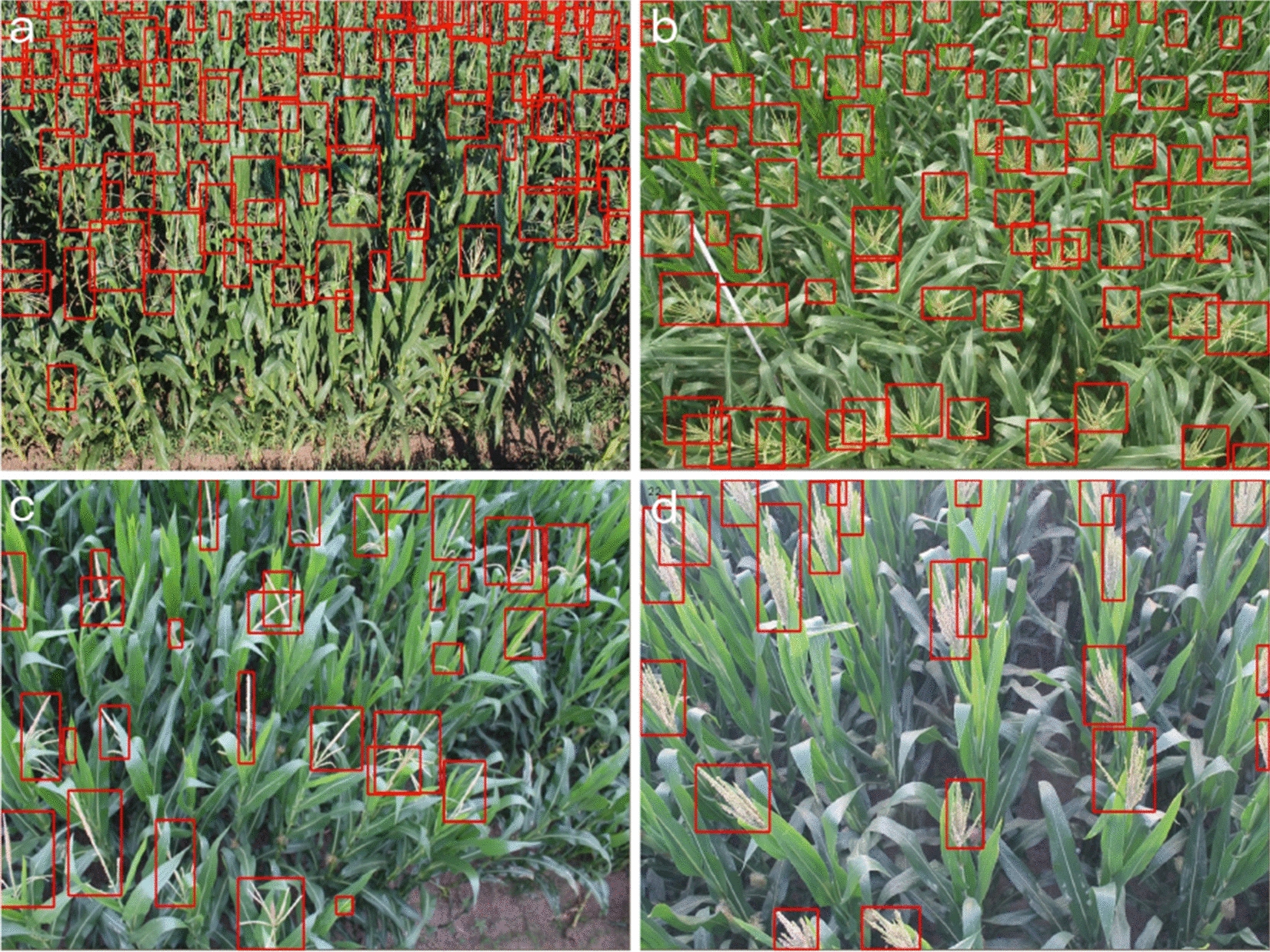
Example images in the MTDC dataset with bounding box annotations. Images are from the **a** Taian2011_2, **b** Taian2010_2, **c** Jalaid2015_2 and **d** Gucheng2014 sequences, respectively

For maize tassel detection, the MTC dataset with only point annotations is insufficient to provide enough information to train a robust deep learning model. Therefore, we re-annotate each tassel with bounding box. In particular, for each tassel, we maintain the center of the bounding box consistent with the dot annotation in MTC dataset. To make a difference, we term MTC dataset with bounding box annotation MTDC dataset. The total number of bounding boxes in the MTDC dataset is 13, 562, and the statistic information of bounding boxes is shown in Fig. 1. Compared with MTC, MTDC can provide more information, such as tassel size, accurate position. Figure 2 shows four example images with bounding box annotations on the MTDC dataset.

To our knowledge, there is only one public dataset for maize tassel detection also released by our team [9]. Compared with the published one, our MTDC dataset includes more genotypes and scenes, and it is more challenging.

### Considered methods

In this section, we first introduce feature pyramid network (FPN) [38] , the feature extractor of Faster R-CNN [22] and RetinaNet [34]. Following it, the details of considered methods are illustrated.

#### Feature pyramid network

Feature representation plays an important role for object detection. To deal with multiscale object representation, feature pyramid is a basic component. However, due to its recent deep learning based object detectors have ignored pyramid representations, in part because they are computation and memory intensive. To make the best of feature pyramids in object detection, Lin et al. [38] proposed the Feature Pyramid Network (FPN) by exploiting the inherent multi-scale, pyramid hierarchy of convolutional network with marginal extra cost.

**Fig. 3:**
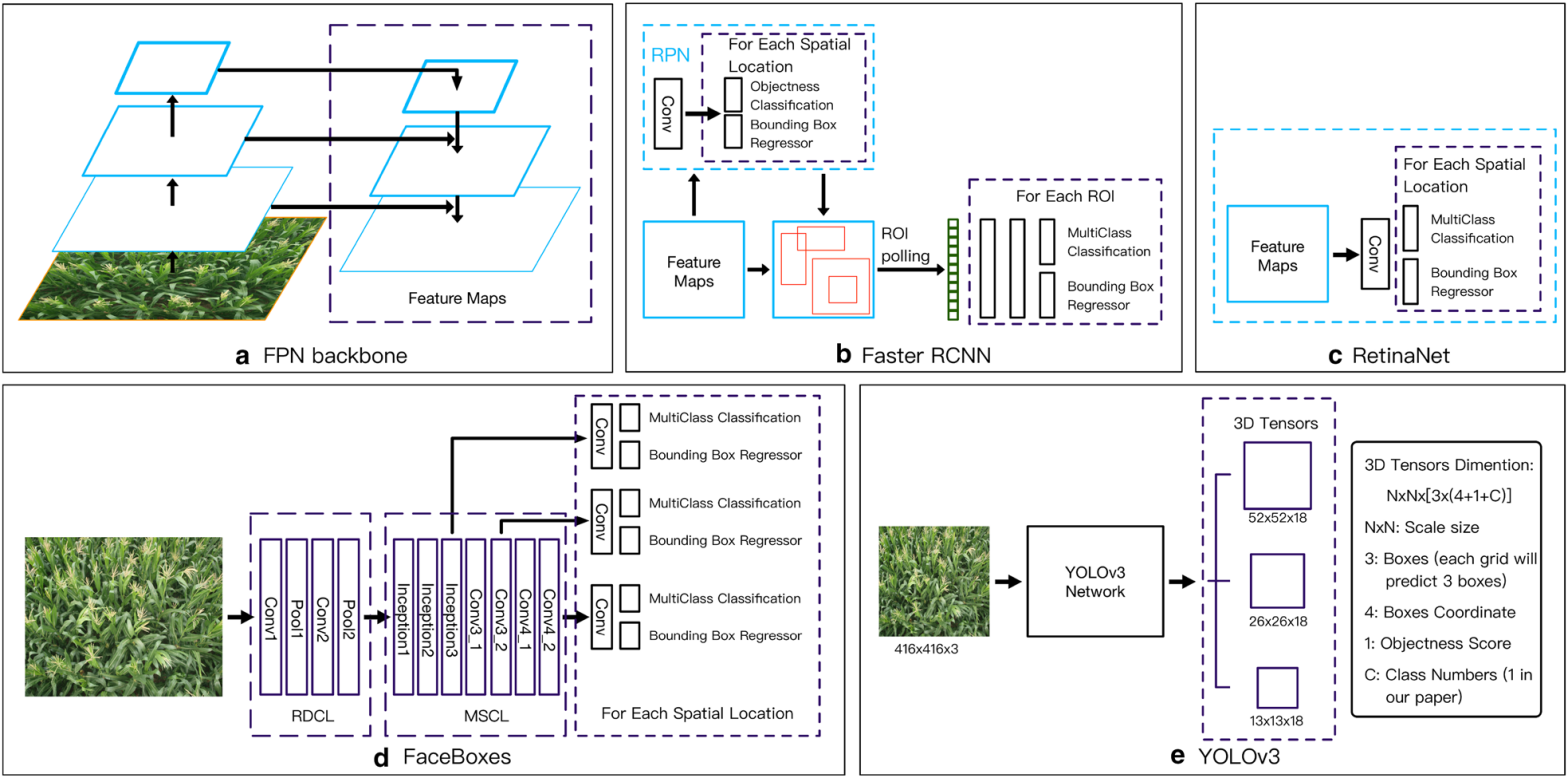
Detection Framework Overview. **a** The Feature Pyramid Network (FPN) [38] used as backbone in Faster R-CNN [22] and RetinaNet [34]. **b**, **c** are diagrams of Faster R-CNN [22] and RetinaNet [34] respectively. **d**, **e** are pipelines for FaceBoxes [36] and YOLOv3 [35] respectively

In brief, FPN is built upon a standard convolutional network by adding a top-down pathway and lateral connections. The bottom-up pathway is the feedforward computation of the backbone ConvNet, and it can compute a feature hierarchy. The top-down pathway upsamples spatially coarser but semantically stronger feature maps from higher pyramid levels to hallucinate higher resolution features. Then feature maps of the same spatial size from bottom-up pathway and top-down pathway are fused with lateral connections. In this way, a rich and multi-scale feature pyramid can be constructed from a single-scale image of arbitrary size(as shown in Fig. 3a). Each level of pyramid can be used for detecting objects at different scales. Following [38], we have built FPN based on ResNet34 architecture [39]. We construct a pyramid with levels $P_3$ through $P_7$, where *l* denotes pyramid level ($P_l$ has resolution $2^l$ lower than the input). The channel number of all pyramid levels is set to 256 as in [38]. The construction of FPN in this paper generally follows [38] with a few modest differences, more details are shown in the reference [38].

#### Faster R-CNN

As a typical two-stage object detection algorithm, Faster R-CNN [22] has been widely applied in many fields since it was proposed. As shown in Fig 3b, based on the extracted feature maps, a region proposal network (RPN) is constructed to generate confident proposal for multi-classification and bounding box refinement.

More precisely, RPN first generates a dense grid of anchor regions (candidate bounding boxes) with specified sizes and aspect ratios over each spatial location of the feature maps. According to intersection over union (IOU) ratio with the ground truth object bounding boxes, an anchor will be assigned with a positive or negative label On top of the feature maps, a shallow CNN is built to judge whether an anchor contains an object and predict an offset for each anchor. Then anchors with high confidence are rectified by the offset predicted in RPN. Then the corresponding features of each anchor will go through a RoI pooling layer, a convolution layer and a fully connected layer to predict a specific class as well as refined bounding boxes. Following current state-of-the-art object detectors [40], we adopt RoIAlign for the RoI pooling layer instead of RoIPool in [22].

In this paper, areas of anchors are from $32^2$ to $512^2$ on levels $P_3$ to $P_7$, respectively. Following [38], three ratios {1:2, 1:1, 2:1} are used to generate anchors. For denser scale coverage, we add anchors with sizes $\{2^0, 2^{1/3}, 2^{2/3}\}$ of the original three aspect ratio anchors at each level additionally. There are $A=9$ anchors in all per level which cover the scale range 32–813 pixels with respect to the input image. As for the configuration of RPN and the second stage, we follow [22].

#### RetinaNet

Different from Faster R-CNN, RetinaNet is a single, unified framework consisting of a CNN backbone and two task-specific subnetworks (as shown in Fig. 3c). Consistent with Faster R-CNN, RetinaNet also adopts ResNet34 based FPN as the backbone to extract feature maps. At each spatial location of extracted feature maps, anchors are with the same configuration as described in *Faster R-CNN*. As for the two task-specific sub-networks, both of them are constructed on top of feature maps with simple convolution operation, the former performing object classification and the latter regressing the position of bounding box.

Moreover, to deal with extreme foreground-background class imbalance during training, the Focal Loss was proposed (for two stage detectors, most of negative proposals are filter by the RPN, so this class imbalance almost does not exist). To address the class imbalance issue, the Focal Loss is modified from the standard cross entropy, which can down-weight the loss assigned to well-classified examples. Supervised by Focal Loss, RetinaNet can achieve significant improvement on generic object detection benchmarks. The definition of focal loss is:

1 $$\begin{aligned} FL(p_{t}) = -{\alpha }_t(1 - p_{t})^{\gamma }\log (p_t) \end{aligned}$$

where $\alpha _t$ and $\gamma$ are hyperparameters. More details of Focal Loss can be referred to [34].

#### FaceBoxes

Maize tassel detection only involves one target class, which is similar to face detection. So we take FaceBoxes [36] into account in our evaluation. FaceBoxes is a one-stage object detector as figured in Fig. 3d. To maintain high performance in real-time, a lightweight yet powerful network structure was designed for FaceBoxes. Furthermore, a new anchor densification strategy was proposed to make different types of anchors have the same density on image.

In detail, the network structure consists of the Rapidly Digested Convolutional Layers (RDCL) and the Multiple Scale Convolutional Layers (MSCL) as shown in Fig. 3d. RDCL is composed of two convolution layers and two pooling layers, which can rapidly shrink the spatial size of input image. What’s more, C.ReLu activation function is used to reduce the number of output channels. On top of RDCL, MSCL was built to extract features at different spatial size. Moreover, convolutional layers are used to predict class confidence and the position offset for anchors sampled on these features. Considering the fact that anchors with small spatial size is sparse than big one, a new anchor densification strategy was proposed to eliminate this imbalance by increasing anchors with small sizes.

#### YOLOv3

Apart from RetinaNet, YOLOv3 [35] is another state-of-the-art one-stage object detector. The pipeline of YOLOv3 is similar to RetinaNet, but the backbone of YOLOv3 is DarkNet-53 based FPN and the feature pyramid only contains 3 level. Although 9 kinds of anchor are used in YOLOv3, there are only 3 for each level of the pyramid according to the spatial size of feature map. What’s more, different from RetinaNet which directly regresses the offsets of anchors, YOLOv3 predicts offsets of each anchor related to the grid that the center of this anchor belongs to. For each anchor, an object score is predicted except the class probability.

#### TasselNet

**Fig. 4:**
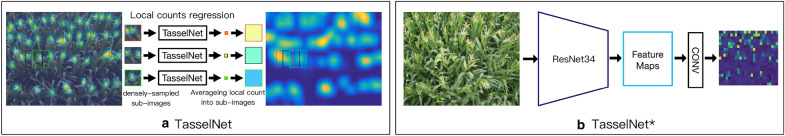
Counting Framework Overview. **a** shows pipeline of the original TasselNet in [6]. **b** is our implementation of TasselNet based on ResNet34 with an entire image as input, we call it $\hbox {TasselNet}^*$

To compare detection and counting methods on maize tassels, we make use of the state-of-the-art tassel counting model TasselNet [6] in this paper. For the sake of fairness, we re-implement TasselNet based on ResNet34, which is named $\hbox {TasselNet}^*$. The pipelines of TasselNet and $\hbox {TasselNet}^*$ are shown in Fig 4, respectively.

TasselNet is a local count regression network composed of deep convolutional neural networks (CNNs). As per Fig 4a, the inputs of TasselNet are sub-images densely sampled from the raw image, and outputs are local counts regressed for each sub-image. At training stage, typical loss functions in regression problems can be used to supervise the network. During the prediction, we can obtain the output density map of the input image by averaging predicted local counts into sub-images. As described in [6], feature extractor has a significant influence on the performence of TasselNet, but original TasselNet adopts VGG16 as its backbone. Here we reconstruct TasselNet with a backbone of ResNet34 and term it $\hbox {TasselNet}^*$ (as shown in Fig. 4b). Noting that $\hbox {TasselNet}^*$ takes an entire image as input instead of sub-images so that speed comparison with Faster R-CNN and RetinaNet is more compellent. $\hbox {TasselNet}^*$ is supervised by $l_1$ Loss at the training stage, and during the prediction, we also feed an entire image to $\hbox {TasselNet}^*$ to obtain the final count directly.

## Evaluation methodology

To thoroughly evaluate the aforementioned algorithms, we design our experiments as follows. Firstly, the implementation details and evaluation metrics are described. Secondly, the evaluated terms are illustrated with some analysis. Finally, summaries are made.

### Implementation details and evaluation metrics

#### Implementation details

Apart from YOLOv3 (based on DarkNet [41]), implementations of all algorithms are based on publicly available PyTorch [42]. All experiments are conducted on the MTDC dataset on a platform of a single Nvidia GeForce GTX TITAN XP GPU (12G). Training set and validation set are mixed together for model learning. Aside from RetinaNet, the other three detectors all adopt Softmax Loss to supervise classification. $Smooth-L_1$ is used in Faster R-CNN, RetinaNet and FaceBoxes for bounding box regression while square error loss is used in YOLOv3.

For a fair comparison, $\hbox {TasselNet}^*$, Faster R-CNN and RetinaNet employ the same data augmentation and network initialization. In particular, for each input image, we first resize it with a minimal side equalling to or greater than 608 and a maximal side equalling to or less than 1024, and then padding it so that width and height can be divided by 32. To avoid overfitting, images are random flipped with a probability of 0.5. As for initialization, ResNet34 backbone is pretrained on ImageNet [43] and other parameters are initialized with the Xavier method [44]. All three methods are optimized with Adam [45] and the BatchNorm layers are freezed. Hyperparameters of these methods are illustrated as follows:

- *Faster R-CNN*: The initial learning rate is 1e–4 and is divided by 10 at the 80th, 160th and 240th epoch, respectively. We set weight decay to 1e–5 and the maximal training epoch to 300 with batch size of 8. The other configurations follow [22].
- *RetinaNet*: We initialize learning rate with 1e–5 and reduce it by a factor of 0.1 when a metric has stopped improving. And we adopt a batch size of 8, a maximum epoch of 300 and a weight decay of 1e–5. For hyperparameters of Focal Loss, we set $\alpha$ to 0.25 and $\gamma$ to 2.0.
- $\hbox {TasselNet}^*$: We set the Gaussian kernel parameter $\sigma = 6$ during generating density map and local patch size to 32 which is equal to the downsampling stride of ResNet34 backbone. MSE loss is used to supervise $\hbox {TasselNet}^*$. Initial learning rate is 1e–6 and drops at 40th and 70th epoch, and weight decay is 2e–5. Because $\hbox {TasselNet}^*$ has a quick convergence compared to Faster R-CNN and RetinaNet, we only train it with 100 epochs. The batch size is set to 1 for training stability.

We adopt the same data augmentation and hard negative mining strategies as in [36] during training FaceBoxes. Stochastic gradient descent (SGD) is used to optimize the parameters of the network randomly initialized with the Xavier [44] method. The batch size, momentum, weight decay and maximal epoch are set to 32, 0.9, 5e–4, 300, respectively. The learning rate is initialized with 1e–3 and divided by 10 at the 200th and 250th epoch, respectively.

We train YOLOv3 on the MTDC dataset exactly following [35]. The DarkNet-53 backbone is initialized by an ImageNet pretrained model, and we turn random resizing on with the batch size of 4 as well as a maximal epoch of 300.

#### Evaluation metrics

The mean absolute error (MAE), the root mean squared error (MSE) and the relative RMSE (rRMSE) are used as the evaluation metrics to assess the counting performance. They take the forms:

2 $$\begin{aligned} MAE= & {} \frac{1}{N}\sum _1^N|t_i-c_i|\,, \end{aligned}$$

3 $$\begin{aligned} MSE= & {} \sqrt{\frac{1}{N}\sum _1^N(t_i-c_i)^2}\,, \end{aligned}$$

4 $$\begin{aligned} rRMSE= & {} \sqrt{\frac{1}{N}\sum _{i=1}^N\left( \frac{t_i-c_i}{t_i}\right) ^2}\,, \end{aligned}$$

where *N* denotes the number of test images, $t_i$ is the ground truth count for the *i*-th image (computed by summing over the whole density map), and $c_i$ is the inferred count for the *i*-th image. As small $t_i$ may lead to large bias on *rRMSE*, here we only take images with $t_i \le 15$ into account. *MAE* quantifies the accuracy of the estimates, *MSE* assesses the robustness of the estimates, and *rRMSE* can justify the high degree of accuracy. The lower these three measures are, the better the counting performance is. The mean average precision (mAP) metric is also used to evaluate performance of detection method. The higher mAP is, the better the detection performance is. We also take inference speed into account with the frame per second (fps) metric.

### Results and analysis

#### Confidence threshold

**Fig. 5:**
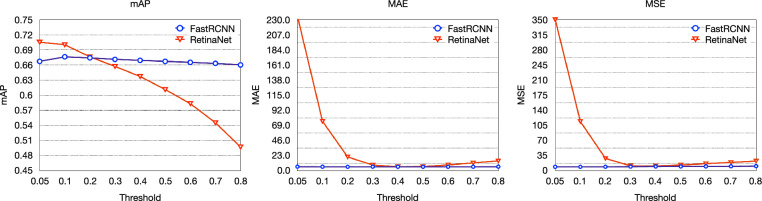
Comparisons of different confidence thresholds for maize tassel detection and counting on the MTDC dataset between Faster R-CNN (blue lines) and RetinaNet (red lines)

The choice of confidence threshold plays an important role in object detection methods, as it determines whether a bounding box includes object or not. Here we first compare different confidence thresholds for maize tassel detection and counting on the MTDC dataset between Faster R-CNN and RetinaNet. Quantitative results are shown in Fig. 5. Blue and red lines indicate Faster R-CNN and RetinaNet, respectively.

According to Fig. 5, we can see that RetinaNet is very sensitive to confidence threshold. With low thresholds, RetinaNet can get high mAP (higher better) but high MAE, MSE (lower better). With increased thresholds, MAE and MSE of RetinaNet firstly decrease rapidly and then increase slightly but mAP keeps decreasing. Different from RetinaNet, Faster R-CNN shows robustness to the confidence threshold as mAP, MAE and MSE almost remain unchanged. The fundamental reason for this is that the first stage of Faster R-CNN can filter many false bounding box before the classification of second stage while RetinaNet has to classify all anchors.

In following experiments, confidence threshold of Faster R-CNN and RetinaNet are set to 0.2 and 0.4 for a fair comparison.

#### Convergence

**Fig. 6:**
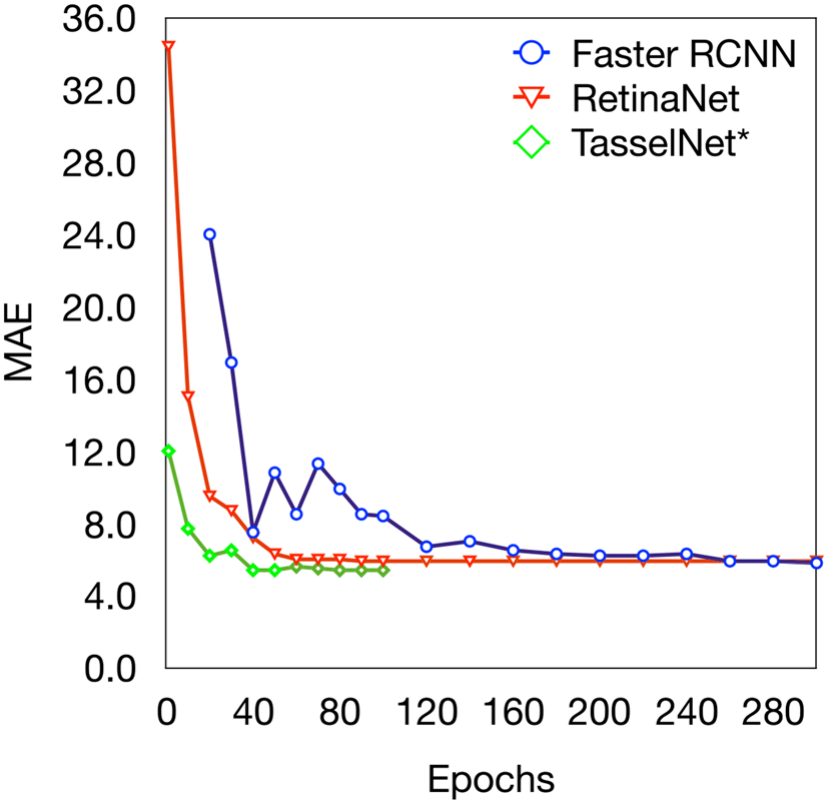
Test errors in terms of MAE versus the number of epochs for Faster R-CNN, RetinaNet and $\hbox {TasselNet}^*$

Here we evaluate the convergence of Faster R-CNN, RetinaNet and $\hbox {TasselNet}^*$ versus the number of epochs. As suggested by Fig. 6, the convergence speed of $\hbox {TasselNet}^*$ is rapid than other two detection methods. Maybe there are many false positive bounding boxes during early period of the training stage of detection algorithms. As focal loss can deal with class imbalance, the learning curve of RetinaNet decreases more quickly and smoothly than Faster R-CNN.

#### Scales

**Fig. 7:**
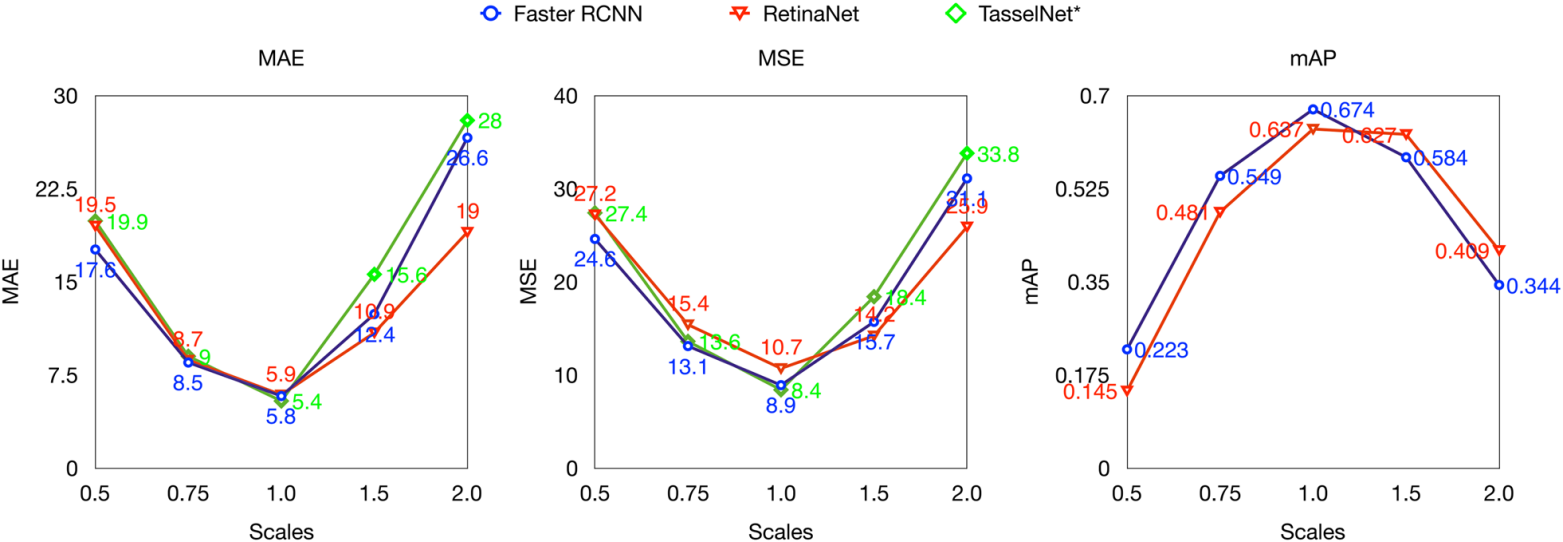
Performance of Faster R-CNN, RetinaNet and $\hbox {tasselNet}^*$ versus scales relative to input image size during training. MAE, MSE and mAP are all taken into account

Figure 7 gives the results with respect to scales relative to input image size during training. It can be observed that $\hbox {TasselNet}^*$ is more sensitive to image size variances than detection methods, i.e., Faster R-CNN and RetinaNet. This is because $\hbox {TasselNet}^*$ has to predict a count for each patch, it may overestimate with larger input image size and underestimate with small input image size. Moreover, we can see that Faster R-CNN shows relative better results with smaller input image size and worse results with larger input image size than RetinaNet.

#### Speed

**Fig. 8:**
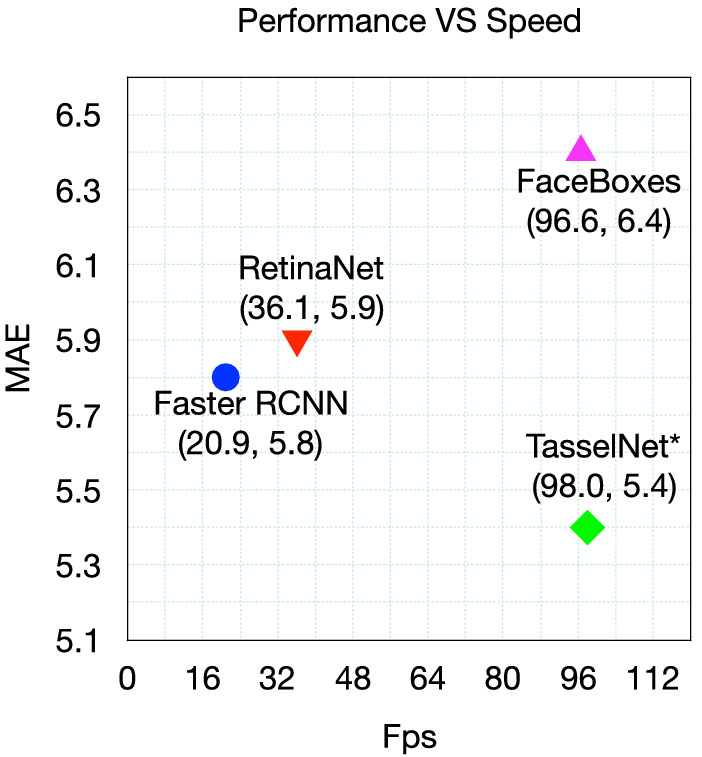
The error in term of MAE versus Speed (fps) on MTDC test set. Faster R-CNN, RetinaNet, FaceBoxes and $\hbox {TasselNet}^*$ are considered

Here we take inference speed into account. Figure 8 shows the error term of MAE versus speed (fps) on the MTDC test dataset. Faster R-CNN, RetinaNet, FaceBoxes and $\hbox {TasselNet}^*$ are considered as they are all implemented with the same Pytorch framework. The input image size of Faster R-CNN, RetinaNet and $\hbox {TasselNet}^*$ are all $640 \times 832$, while the input image size of FaceBoxes is $1024 \times 1024$ (it is trained with this size). We measure speed of all methods on a PC of single Nvidia GeForce GTX TITAN XP GPU (12G). According to Fig. 8, $\hbox {TasselNet}^*$ outperforms other three methods both on speed (fps) and MAE (lower is better). RetinaNet is faster than Faster R-CNN as the second stage of Faster R-CNN is time-consuming, but Faster R-CNN achieves a lower MAE. FaceBoxes can achieve a comparable speed with $\hbox {TasselNet}^*$, this is somewhat expected, i.e., the network of FaceBoxes is specifically designed for real-time application. How to design a faster and better network for detection is still a hot research area.

#### Linear regression coefficients between considered algorithms and manual counts

**Fig. 9:**
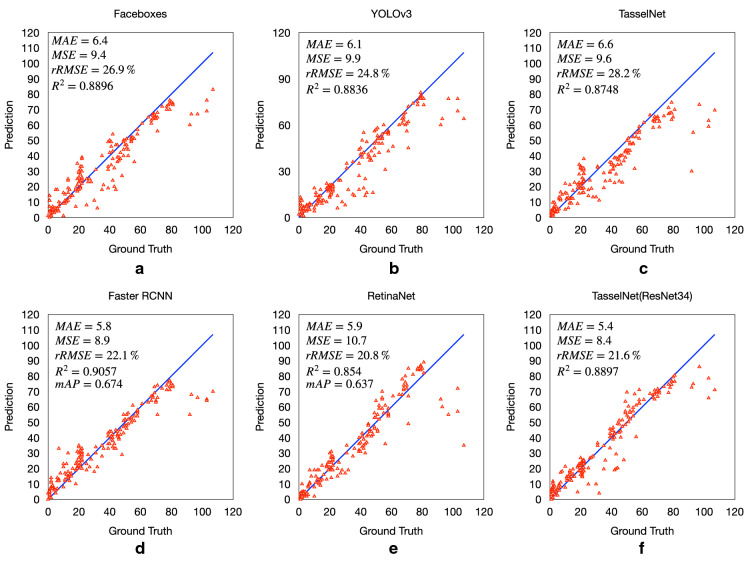
Plots of Manual counting versus different Algorithms counting on MTDC test set. **a** FaceBoxes, **b** YOLOv3, **c** original TasselNet, **d** Faster R-CNN, **e** RetinaNet and **f**$\hbox {TasselNet}^*$

The linear regressions between the manual counting and considered algorithms counting calculated for the MTDC test set are shown in Fig 9. We can observe that, Faster R-CNN and RetinaNet work better than $\hbox {TasselNet}^*$ with small counts but worse with large counts. When there are too many tassels occur in a single image, occlusions between tassels are hard for detection methods, many predicted bounding boxes will be filter by non maximum suppression of detector and may lead to an underestimation. Comparing original TasselNet and our $\hbox {TasselNet}^*$, we can find that a better backbone can achieve lower MAE and rRMSE error. Moreover, original TasselNet tends to underestimate the tassel count while our $\hbox {TasselNet}^*$ does not, which may benefit from an entire image as input, i.e., $\hbox {TasselNet}^*$ can catch extra global information.

#### Comparison with the state-of-the-art

We also compare aforementioned methods with several well-established baseline approaches on the MTDC test set,and a brief introduction is illustrated as follow:

- *JointSeg* [46]: JointSeg is the state-of-the-art tassel segmentation method. Based on the segmentation results, we can easily get object counts. To reduce noise interference, some morphological operations are performed as post-correction. This approach can be viewed as a counting-by-segmentation baseline.
- *mTASSEL* [9]: mTASSEL is a specifically designed detection method for maize tassel. It makes use of multi-view representations to characterise the visual characteristics of tassels and achieved state-of-the-art detection result. This is a counting-by-detection baseline.
- *GlobalReg* [47]: GlobalReg is a state-of-the-art crowd counting approach that directly regress the global count of a image. It adopted a pretrained model to extract holistic image representation and mapped these global feature into object count by ridge regression. This can be viewed as a global counting-by-regression baseline.
- *DensityReg* [48]: DensityReg proposed to predict idea of density map regression that predicts a count for every pixel by optimising the MESA distance. It is a global density-based counting-by-regression baseline.
- *Counting-CNN (CCNN)* [49]: CCNN is a state-of-the-art object counting method that regresses the local density map with a AlexNet-like CNN architecture. This is a local density-based counting-by-regression baseline.

**Fig. 10:**
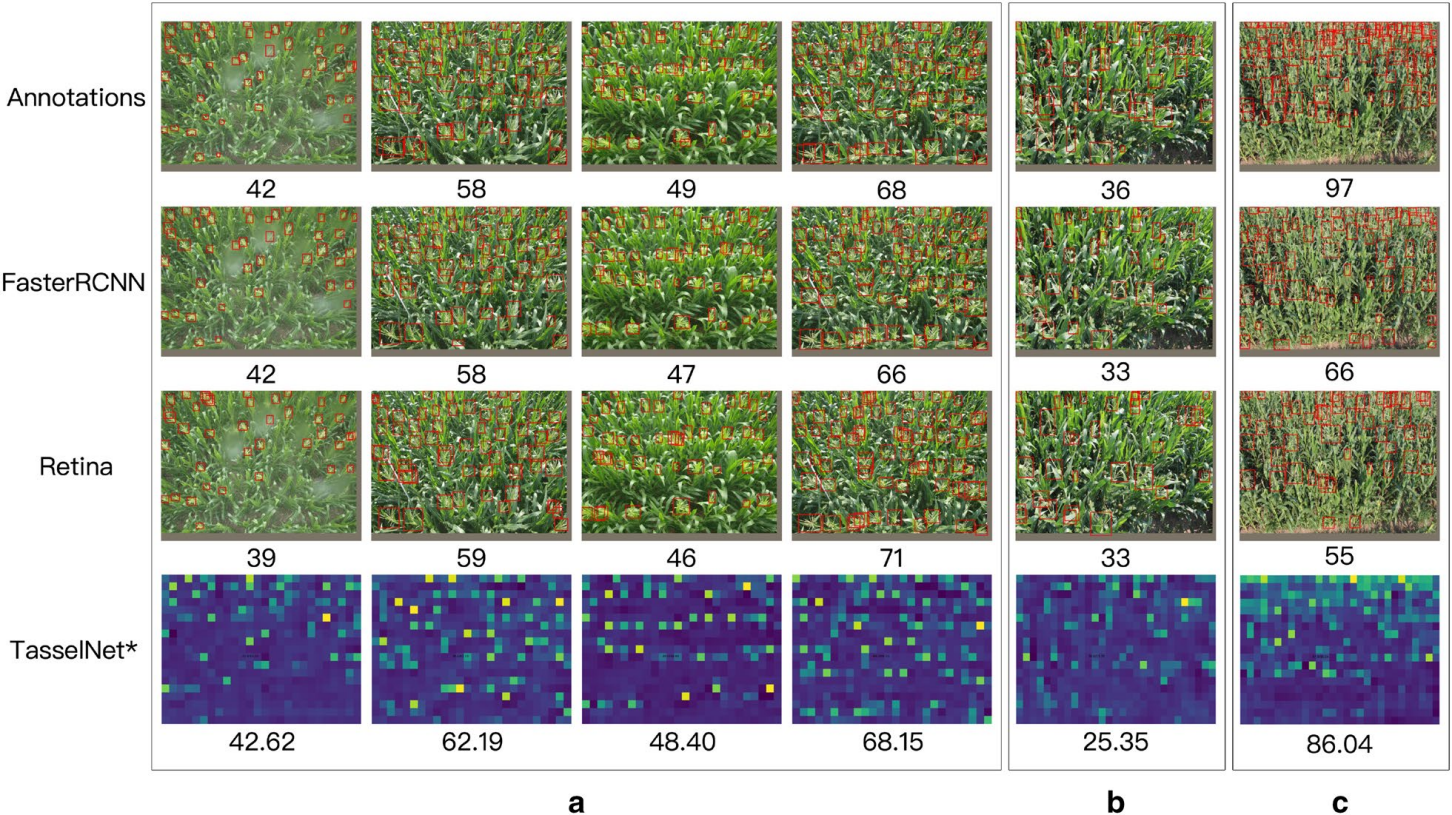
Qualitative results of ground truth bounding boxes and the predicted results of Faster R-CNN, RetinaNet and $\hbox {TasselNet}^*$, respectively. The number shown below each sub-figure denotes the tassel count over the predicted results

Table 2 shows the quantitative results and Fig. 10 shows the qualitative results. The following observations can be made from Table 2 and Fig. 10:

- The state-of-the-art detection methods can achieve comparable results with the best regression based counting algorithms. In most test sequences, Faster R-CNN can achieve lower *MAE* and *MSE* than $\hbox {TasselNet}^*$.
- Compared with the best one stage detection methods, i.e. RetinaNet, the best two stage detection method Faster R-CNN can obtain lower errors and better bounding boxes.
- $\hbox {TasselNet}^*$ outperforms TasselNet in 7 out of 8 testing sequences, and achieves the best overall counting performance—the lowest *MAE* and *MSE* errors. This is somewhat expected, i.e., advanced architecture can achieve better counting performance.
- Almost all methods can obtain a higher *MAE* (and *MSE*) in Jalaid2015_2 and Jalaid2015_3 sequence. And we find that the category of tassel in these two sequences are quite different other sequences, and the training set has a different distribution with test set. So one may consider to alleviate these issues by adding more extra training data or trying domain adaptation [50, 51].
- Qualitative results in Fig. 10a shows that Faster R-CNN, RetinaNet and $\hbox {TasselNet}^*$ all can estimate reasonable approximations to the ground truth counts. However, $\hbox {TasselNet}^*$ works poorly if the scales of tassels in an image vary a lot (as shown in Fig. 10b). This is consitent with the observation made in [7]. A main reason is that the gaussian kernel used to generate the density map is a constant which can not deal with scale variances. By contrast, Faster R-CNN and RetinaNet are more robust to scale variance because they are trained with the supervision of bounding boxes. But as shown in Fig. 10c, Faster R-CNN and RetinaNet tend to underestimate the count when tassels are occluded by each other or the size of tassels is too small. Because many object bounding boxes will be filtered by non-maximal suppression operation even boxes have high confident scores. It should be noted that $\hbox {TasselNet}^*$ performs well in this crowded scene. As Faster R-CNN (or RetinaNet) and $\hbox {TasselNet}^*$ can complement each other, it may be possible to improve performance by combining them. We leave these explorations open at present.

**Table 2:** Mean absolute errors (MAE) and mean squared errors (MSE) for maize tassels counting on the test set of MTC dataset

Method	Sequences																Overall	
	Zhengzhou2011		Taian2010_2		Taian2011_2		Taian2012_2		Taian2013_2		Gucheng2014		Jalaid2015_2		Jalaid2015_3			
	MAE	MSE	MAE	MSE	MAE	MSE	MAE	MSE	MAE	MSE	MAE	MSE	MAE	MSE	MAE	MSE	MAE	MSE
JointSeg	20.9	23.2	46.6	47.9	16.4	19.7	25.1	29.8	6.5	8.0	7.3	10.5	27.8	29.1	53.2	61.3	24.2	31.6
mTASSEL	9.8	14.9	18.6	22.1	11.6	12.7	5.3	7.8	13.1	16.6	31.1	35.3	16.2	18.0	46.6	51.0	19.6	26.1
GlobalReg	19.0	21.5	23.0	24.7	14.1	16.8	13.5	15.7	19.6	25.2	19.5	21.7	11.2	13.7	42.1	45.4	19.7	23.3
DensityReg	16.1	20.2	9.9	10.7	9.2	11.7	10.8	12.7	20.2	23.7	9.4	10.5	**7.2**	**7.9**	23.5	26.9	11.9	14.8
CCNN	21.3	23.3	28.9	31.6	12.4	16.0	12.6	15.3	18.9	23.7	21.6	24.1	9.6	12.4	39.5	46.4	21.0	25.5
TasselNet	4.9	6.1	5.2	6.6	**2**.**5**	**2**.**9**	4.8	5.8	4.0	5.0	5.3	6.5	16.0	16.6	20.7	25.2	6.6	9.6
$\hbox {TasselNet}^*$	***3***.***0***	***3***.***4***	**3**.**2**	4.2	3.2	3.7	5.2	7.1	***3.5***	4.8	***3.4***	***4.0***	18.3	19.2	**16.3**	**19**.**8**	**5**.**4**	**8**.**4**
FaceBoxes	7.3	8.4	3.7	***4.4***	3.3	4.2	3.3	4.4	4.9	6.3	4.1	6.1	18.5	19.2	***19.3***	***22.1***	6.4	9.4
YOLOv3	4.3	5.7	5.0	6.6	***2***.***9***	3.9	***3.0***	***4.0***	4.5	6.0	**2**.**3**	**2**.**8**	20.3	21.7	24.1	25.9	6.1	9.9
RetinaNet	3.3	4.3	5.6	6.4	4.4	6.6	3.3	***4.0***	**3**.**1**	**4**.**0**	4.2	5.1	***7.6***	***8.1***	26.5	32.9	5.9	10.7
Faster R-CNN	**2**.**6**	**3**.**1**	**3**.**2**	**4**.**0**	***2***.***9***	***3***.***4***	**2**.**6**	**3**.**2**	3.6	***4.4***	7.6	9.0	8.6	9.6	21.3	25.5	***5.8***	***8.9***

The two lowest error are shown in bold and bold italic

### Summaries

Here we evaluate some state-of-the-art object detection and object counting methods on our proposed MTDC dataset from different aspects, and our findings can be summarised as follows:

1. Faster R-CNN is more robust than RetinaNet for object counting, as the performance of the former one nearly remain unchanged with different confidence thresholds while the latter varies a lot.
2. Regression based counting methods converge faster than object detection based methods. In detection, RetinaNet converges faster than Faster R-CNN.
3. Compared with detection based methods, regression based counting methods are more sensitive to object scales as the latter are trained in a way independent of scales.
4. On the aspect of speed, regression based counting methods are faster than detection-based ones,but it is possible to accelerate object detectors by designing a lightweight network.
5. With a stronger backbone, TasselNet can achieve better performance and extra information can benefit regression based counting methods.
6. The state-of-the-art detection methods can actually achieve comparable results with the state-of-the-art counting methods based on regression.
7. Detection based methods tend to underestimate the number of tassels in crowded scene because of heavy occlusions.
8. Apart from the count, detection based methods can extract more information about tassels in an image, such as position and size.

According to our observations, we suggest some possible solutions to help deal with maize-tassel-like in-field counting problems:

1. Design a lightweight network to accelerate current counting algorithm so that it can run on a CPU.
2. Try fusing local and global information to improve regression-based counting methods.
3. Joint detection and regression based algorithms that make full use of their advantages in different circumstances.
4. Try the idea of counting by regression in crowd scene and counting by detection when the scales of tassels change a lot.
5. Try the idea of data synthesis to augment training data.
6. Try to use domain adaptation [50, 51] to fill the differences between sequences, e.g. domain adaptive Faster R-CNN [52].

## Conclusions

In this paper, we evaluate some state-of-the-art object detection algorithms for in-field counting of maize tassels. We create the MTDC dataset by supplementing bounding box annotations to the MTC dataset so that we can train detectors on it. We fairly compare these state-of-the-art object detection methods and regression-based counting approaches in robustness, speed, performance and other aspects. Summaries of the advantages and limitations of each method are provided. Results show that different detection frameworks all provide acceptable accuracy in maize tassel detection. Our evaluations thus can be a useful reference for practitioners to save their time when choosing a plant counting model to deal with a similar plant detection problem. In addition, maize tassels are typical non-rigid objects. Our work provides a dataset and several strong baselines for researchers who are interested in improving the accuracy of non-rigid object detection. Furthermore, TasselNet reports comparable results. This delivers a message that regression-based methods may be a better choice than detection ones when only the population of instances is of interest, because regression-based methods only require less expensive dotted annotations and take less training and inference time.

We also point out some possible directions to improve tassel counting with object detection and regression based methods. We hope our work can facilitate the popularization of computer vision technologies in plant science.

## Data Availability

The MTDC dataset and other supporting materials are made available at https://git.io/MTDC.

## References

[1] Tardieu F, Cabrera-Bosquet L, Pridmore T, Bennett M (2017). Plant phenomics, from sensors to knowledge. Curr Biol.

[2] Pourreza A, Lee WS, Etxeberria E, Banerjee A (2015). An evaluation of a vision-based sensor performance in huanglongbing disease identification. Biosyst Eng.

[3] Gómez-Flores W, Garza-Saldaña JJ, Varela-Fuentes SE (2019). Detection of huanglongbing disease based on intensity-invariant texture analysis of images in the visible spectrum. Comput Electron Agric.

[4] Tello J, Montemayor MI, Forneck A, Ibáñez J (2018). A new image-based tool for the high throughput phenotyping of pollen viability: evaluation of inter- and intra-cultivar diversity in grapevine. Plant Methods.

[5] Guerrero JM, Pajares G, Montalvo M, Romeo J, Guijarro M (2012). Support vector machines for crop/weeds identification in maize fields. Expert Syst Appl.

[6] Lu H, Cao Z, Xiao Y, Zhuang B, Shen C (2017). Tasselnet: counting maize tassels in the wild via local counts regression network. Plant Methods.

[7] Madec S, Jin X, Lu H, Solan BD, Liu S, Duyme F, Heritier E, Baret F (2019). Ear density estimation from high resolution rgb imagery using deep learning technique. Agric For Meteorol.

[8] Hasan MM, Chopin JP, Laga H, Miklavcic SJ (2018). Detection and analysis of wheat spikes using convolutional neural networks. Plant Methods.

[9] Lu H, Cao Z, Xiao Y, Fang Z, Zhu Y, Xian K (2015). Fine-grained maize tassel trait characterization with multi-view representations. Comput Electron Agric.

[10] Guo W, Fukatsu T, Ninomiya S (2015). Automated characterization of flowering dynamics in rice using field-acquired time-series RGB images. Plant Methods.

[11] Sakamoto T, Gitelson AA, Nguy-Robertson AL, Arkebauer TJ, Wardlow BD, Suyker AE, Verma SB, Shibayama M (2012). An alternative method using digital cameras for continuous monitoring of crop status. Agric For Meteorol.

[12] Ye M, Cao Z, Yu Z. An image-based approach for automatic detecting tasseling stage of maize using spatio-temporal saliency. In: Proceedings of the Eighth International Symposium on Multispectral Image Processing and Pattern Recognition; 2013, p. 89210. International Society for Optics and Photonics. 10.1117/12.2031024.

[13] Zhu Y, Cao Z, Lu H, Li Y, Xiao Y (2016). In-field automatic observation of wheat heading stage using computer vision. Biosyst Eng.

[14] Bannayan M, Sanjani S (2011). Weather conditions associated with irrigated crops in an arid and semi arid environment. Agric For Meteorol.

[15] Li Q, Dong B, Qiao Y, Liu M, Zhang J (2010). Root growth, available soil water, and water-use efficiency of winter wheat under different irrigation regimes applied at different growth stages in north china. Agric Water Manage.

[16] Qiongyan L, Cai J, Berger B, Okamoto M, Miklavcic SJ (2017). Detecting spikes of wheat plants using neural networks with laws texture energy. Plant Methods.

[17] Aich S, Stavness I. Leaf counting with deep convolutional and deconvolutional networks. In: Proc. IEEE International Conference on Computer Vision Workshops (ICCVW); 2017, p. 2080–9. 10.1109/ICCVW.2017.244.

[18] Kumar JP, Domnic S (2019). Image based leaf segmentation and counting in rosette plants. Inform Process Agric.

[19] Rizon M, Yazid H, Saad P, Shakaff AYM, Saad AR, Sugisaka M, Yaacob S, Mamat MR, Karthigayan M. Object detection using circular hough transform 2005.

[20] Rahnemoonfar M, Sheppard C (2017). Deep count: fruit counting based on deep simulated learning. Sensors.

[21] Girshick R, Donahue J, Darrell T, Malik J. Rich feature hierarchies for accurate object detection and semantic segmentation. In: Proc. IEEE Conference on Computer Vision and Pattern Recognition (CVPR); 2014, p. 580–7. 10.1109/CVPR.2014.81.

[22] Ren S, He K, Girshick R, Sun J (2017). Faster r-cnn: towards real-time object detection with region proposal networks. IEEE Trans Pattern Anal Mach Intell.

[23] Ubbens J, Cieslak M, Prusinkiewicz P, Stavness I (2018). The use of plant models in deep learning: an application to leaf counting in rosette plants. Plant Methods.

[24] Tsaftaris S, Scharr H. Computer vision problems in plant phenotyping, CVPPP; 2014. https://www.plant-phenotyping.org/CVPPP2014.

[25] Tsaftaris S, Scharr H, Pridmore T. Computer vision problems in plant phenotyping, CVPPP; 2015. https://www.plant-phenotyping.org/CVPPP2015.

[26] Tsaftaris S, Scharr H, Pridmore T. Computer vision problems in plant phenotyping, CVPPP; 2017. https://www.plant-phenotyping.org/CVPPP2017.

[27] Tsaftaris S, Scharr H, Pridmore T. Computer vision problems in plant phenotyping, CVPPP; 2018. https://www.plant-phenotyping.org/CVPPP2018.

[28] Tsaftaris S, Scharr H, Pridmore T. Computer vision problems in plant phenotyping, CVPPP; 2019. https://www.plant-phenotyping.org/CVPPP2019.

[29] Lu H, Dai Y, Shen C, Xu S. Indices matter: Learning to index for deep image matting. In: Proc. IEEE International Conference on Computer Vision (ICCV); 2019, p. 3266–75. 10.1109/ICCV.2019.00336.

[30] Lu H, Dai Y, Shen C, Xu S (2020). Index networks. IEEE Trans Pattern Anal Mach Intell.

[31] Girshick R. Fast R-CNN. In: Proc. IEEE International Conference on Computer Vision (ICCV); 2015, p. 1440–8.10.1109/ICCV.2015.169.

[32] Liu W, Anguelov D, Erhan D, Szegedy C, Reed S, Fu C.-Y, Berg A.C. Ssd: Single shot multibox detector. In: Proc. European Conference on Computer Vision (ECCV); 2016, p. 21–37. 10.1007/978-3-319-46448-0_2.

[33] Redmon J, Farhadi A. Yolo9000: Better, faster, stronger. In: Proc. IEEE Conference on Computer Vision and Pattern Recognition (CVPR); 2017, p. 6517–25. 10.1109/CVPR.2017.690.

[34] Lin T.-Y, Goyal P, Girshick R, He K, Dollar P. Focal loss for dense object detection. In: Proc. IEEE International Conference on Computer Vision (ICCV); 2017. 10.1109/iccv.2017.324.

[35] Redmon J, Farhadi A. Yolov3: an incremental improvement; 2018. arXiv preprint arXiv:1804.02767.

[36] Zhang S, Zhu X, Lei Z, Shi H, Wang X, Li S.Z. Faceboxes: a cpu real-time face detector with high accuracy. In: Proc. IEEE International Joint Conference on Biometrics (IJCB); 2017. 10.1109/btas.2017.8272675.

[37] Simonyan K, Zisserman A. Very deep convolutional networks for large-scale image recognition; 2014. CoRR **abs/1409.1556**.

[38] Lin T.-Y, Dollar P, Girshick R, He K, Hariharan B, Belongie S. Feature pyramid networks for object detection. In: Proc. IEEE Conference on Computer Vision and Pattern Recognition (CVPR); 2017. 10.1109/cvpr.2017.106.

[39] He K, Zhang X, Ren S, Sun J. Deep residual learning for image recognition. In: Proc. IEEE Conference on Computer Vision and Pattern Recognition (CVPR); 2016. 10.1109/cvpr.2016.90.

[40] He K, Gkioxari G, Dollar P, Girshick R. Mask r-cnn. In: Proc. IEEE International Conference on Computer Vision (ICCV); 2017. 10.1109/iccv.2017.322.

[41] Redmon J. Darknet: open source neural networks in C. http://pjreddie.com/darknet/ (2013–2016).

[42] Paszke A, Gross S, Chintala S, Chanan G. PyTorch; 2017. https://pytorch.org/.

[43] Deng J, Dong W, Socher R, Li L.-J, Li K, Fei-Fei L. Imagenet: A large-scale hierarchical image database. In: Proc. IEEE Conference on Computer Vision and Pattern Recognition (CVPR); 2009, p. 248–55. 10.1109/CVPR.2009.5206848.

[44] Glorot X, Bengio Y. Understanding the difficulty of training deep feedforward neural networks. In: Proceedings of the Thirteenth International Conference on Artificial Intelligence and Statistics; 2010, p. 249–56.

[45] Kingma DP, Ba J. Adam: a method for stochastic optimization; 2014. arXiv preprint arXiv:1412.6980.

[46] Lu H, Cao Z, Xiao Y, Li Y, Zhu Y (2016). Region-based colour modelling for joint crop and maize tassel segmentation. Biosyst Eng.

[47] Tota K, Idrees H. Counting in dense crowds using deep features. CRCV; 2015.

[48] Lempitsky V, Zisserman A. Learning to count objects in images. In: Advances in neural information processing systems (NIPS); 2010, p. 1324–32. http://papers.nips.cc/paper/4043-learning-to-count-objects-in-images.

[49] Onoro-Rubio D, López-Sastre RJ. Towards perspective-free object counting with deep learning. In: Proc. European Conference on Computer Vision (ECCV); 2016, p. 615–29. Springer. 10.1007/978-3-319-46478-7_38.

[50] Lu H, Cao Z, Xiao Y, Zhu Y (2017). Two-dimensional subspace alignment for convolutional activations adaptation. Pattern Recogn.

[51] Lu H, Zhang L, Cao Z, Wei W, Xian K, Shen C, v. d. Hengel A. When unsupervised domain adaptation meets tensor representations. In: Proc. IEEE International Conference on Computer Vision (ICCV); 2017, p. 599–608.

[52] Chen Y, Li W, Sakaridis C, Dai D, Van Gool L. Domain adaptive faster r-cnn for object detection in the wild. In: Proc. IEEE Conference on Computer Vision and Pattern Recognition (CVPR); 2018, p. 3339–48.

